# Analysis of the Localization of *Schizosaccharomyces pombe* Glucan Synthases in the Presence of the Antifungal Agent Caspofungin

**DOI:** 10.3390/ijms24054299

**Published:** 2023-02-21

**Authors:** Esther San-Quirico, M. Ángeles Curto, Laura Gómez-Delgado, M. Belén Moreno, Pilar Pérez, Juan Carlos Ribas, Juan Carlos G. Cortés

**Affiliations:** Instituto de Biología Funcional y Genómica, Consejo Superior de Investigaciones Científicas (CSIC) and Universidad de Salamanca, 37007 Salamanca, Spain

**Keywords:** fungi, yeast, fission yeast, invasive mycoses, cell wall, glucan, β(1-3)glucan synthase, antifungals, echinocandins, caspofungin, antifungal resistance, cytokinesis, cell lysis, green fluorescent protein

## Abstract

In recent years, invasive fungal infections have emerged as a common source of infections in immunosuppressed patients. All fungal cells are surrounded by a cell wall that is essential for cell integrity and survival. It prevents cell death and lysis resulting from high internal turgor pressure. Since the cell wall is not present in animal cells, it is an ideal target for selective invasive fungal infection treatments. The antifungal family known as echinocandins, which specifically inhibit the synthesis of the cell wall β(13)glucan, has been established as an alternative treatment for mycoses. To explore the mechanism of action of these antifungals, we analyzed the cell morphology and glucan synthases localization in *Schizosaccharomyces pombe* cells during the initial times of growth in the presence of the echinocandin drug caspofungin. *S. pombe* are rod-shaped cells that grow at the poles and divide by a central division septum. The cell wall and septum are formed by different glucans, which are synthesized by four essential glucan synthases: Bgs1, Bgs3, Bgs4, and Ags1. Thus, *S. pombe* is not only a perfect model for studying the synthesis of the fungal β(1-3)glucan, but also it is ideal for examining the mechanisms of action and resistance of cell wall antifungals. Herein, we examined the cells in a drug susceptibility test in the presence of either lethal or sublethal concentrations of caspofungin, finding that exposure to the drug for long periods at high concentrations (>10 µg/mL) induced cell growth arrest and the formation of rounded, swollen, and dead cells, whereas low concentrations (<10 µg/mL) permitted cell growth with a mild effect on cell morphology. Interestingly, short-term treatments with either high or low concentrations of the drug induced effects contrary to those observed in the susceptibility tests. Thus, low drug concentrations induced a cell death phenotype that was not observed at high drug concentrations, which caused transient fungistatic cell growth arrest. After 3 h, high concentrations of the drug caused the following: (i) a decrease in the GFP-Bgs1 fluorescence level; (ii) altered locations of Bgs3, Bgs4, and Ags1; and (iii) a simultaneous accumulation of cells with calcofluor-stained incomplete septa, which at longer times resulted in septation uncoupling from plasma membrane ingression. The incomplete septa revealed with calcofluor were found to be complete when observed via the membrane-associated GFP-Bgs or Ags1-GFP. Finally, we found that the accumulation of incomplete septa depended on Pmk1, the last kinase of the cell wall integrity pathway.

## 1. Introduction

It is estimated that each year 1.6 million people die from and more than 300 million are affected by fungal infections. Systemic fungal infections have emerged as a major problem for healthcare structures [1,2]. Thus, the most at-risk patients are those with primary health problems or those who are immunosuppressed because of AIDS and other illnesses, such as cancer, chronic lung disease, diabetes mellitus, or tuberculosis. Patients in intensive care units, those undergoing invasive medical practices and being treated with broad-spectrum antibiotics, and those undergoing organ transplantation and receiving immunosuppressant drugs are also at great risk [1,3,4]. Consequently, the World Health Organization (WHO) recently published a report with a list of fungal priority pathogens (WHO FPPL) that warns of the growing threat of systemic fungal infections [3]. This problem is further aggravated by the emergence of antifungal resistance and the existence of only four classes of systemic antifungal drugs (azoles, echinocandins, polyenes, and pyrimidines) that can be used in clinical treatments [3,5,6,7].

The fungal cell wall is an external polysaccharidic framework that maintains the internal turgor pressure and, thus, is vital for cell integrity and survival [8,9]. In addition, because the cell wall is not present in infected animal hosts, it is the most suitable target for discovering and developing potential drugs with broad fungicidal activities [10,11,12]. β(1-3)glucan is the most abundant polysaccharide in the fungal cell wall structure. This polysaccharide is synthetized by the glycosyltransferase β(1-3)glucan synthase (βGS), whose catalytic subunit is constituted by the family of integral membrane proteins Bgs/Fks in all fungi studied [12,13,14]. Treatment with molecules that block βGS activity causes osmotic fragility and cell lysis in actively growing cells [15,16,17,18].

The fission yeast *Schizosaccharomyces pombe* has been widely studied as a model for investigating the role of β(1-3)glucan in cell wall synthesis, morphogenesis, and cell integrity [8,9,19]. It contains four essential βGS catalytic subunits, which localize to the plasma membrane of the division site and growing poles: Bgs1 synthetizes the minor linear β(1-3)glucan responsible for the primary septum structure, which is specifically stained with the fluorochrome calcofluor white (CW) and is required for cleavage furrow ingression [20,21,22]. Bgs1 is mostly localized as a ring, which is tightly associated with the contractile actomyosin ring, at the tip of the septum membrane during ingression [21,23,24,25,26]; Bgs2 is required for spore wall maturation [27,28]; the function of Bgs3 remains unknown [29]; and Bgs4 synthetizes the major branched β(1-3)glucan responsible for the maintenance of cell shape and for both septum formation and completion. In contrast to Bgs1 and Bgs3, the function of Bgs4 is essential for cell integrity and, thus, Bgs4 depletion causes cell lysis and cytoplasm release into the medium, both in the growing poles and, to a greater extent, in the cell medial region at the onset of septum degradation and cell separation [30,31,32]. Interestingly, the loss of branched β(1-3)glucan also generates an increased phenotype of uncoupling between a fast ring and membrane ingression and slow primary septum synthesis, giving rise to complete septa that are exclusively stained with CW in the external edges [25,31]. The fission yeast cell wall also contains an essential α(1-3)glucan, which is synthetized by the α(1,3)glucan synthase (αGS) Ags1/Mok1 [33,34,35].

Three classes of antifungals, i.e., lipopeptides (echinocandins), acidic terpenoids (enfumafungin), and glycolipids (papulacandins), target βGS activity and affect cell wall integrity. From these, only the echinocandins caspofungin, micafungin, and anidulafungin have been approved as drugs for treating systemic fungal infections [5,11,36,37]. Recently, the novel echinocandin rezafungin was designated as a Qualified Infectious Disease Product Designation with Fast Track status by the U.S. Food and Drug Administration and was granted Orphan Drug Designation for invasive candidiasis therapy in both the E.U. and U.S. [38]. Echinocandins obstruct the synthesis of β(1-3)-D-glucan through noncompetitive inhibition of βGS activity [39,40,41,42,43]. However, the integral membrane βGS catalytic subunit has not been homogenously purified and, thus, the molecular mechanism of βGS inhibition by echinocandins is not entirely known [13,44,45,46]. The emergence of resistance to βGS antifungals is generally related to point mutations in highly conserved short regions of the Bgs/Fks proteins [30,47,48,49]. In *S. pombe*, the fact that the *bgs4^+^* mutant strains *pbr1-8* and *pbr1-6* exhibit resistance to echinocandins and other classes of βGS antifungals, even in the presence of the wild-type (WT) sequences of *bgs1^+^* and *bgs3^+^*, suggests that the two encoding synthases, Bgs1 and Bgs3, might be naturally resistant to the currently available βGS inhibitors [30]. However, recent findings show that lethal concentrations of anidulafungin and caspofungin produce early cytokinesis arrest in *S. pombe* WT and *pbr1-8* mutant cells, suggesting at least a partial combined inhibition of several βGSs [50].

Both the conservation of β(1-3)glucan synthesis and the corresponding βGS enzymes across the fungi kingdom and the presence of three essential βGS catalytic subunits exhibiting both differential antifungal susceptibility and non-redundant essential roles in β(1-3)glucan synthesis, together with the absence of cell wall chitin and, therefore, chitin-derived compensatory mechanisms, make *S. pombe* an ideal tool for studying resistance and the mechanism of action of echinocandins despite its lack of pathogenicity [30,50]. The GS subunits Bgs1, Bgs3, Bgs4, and Ags1 are each essential for cell viability as a result of their different impacts on cytokinesis and cell integrity. Thus, we compared the effect of different concentrations of caspofungin on the localization of the four essential GSs. For that purpose, *S. pombe* strains containing each of the four GSs tagged with the green fluorescence protein (GFP-GSs) were imaged using CW and GFP fluorescence microscopy during early times of growth in the presence of different concentrations of caspofungin. Other studies with echinocandins analyzed the long-term effect of antifungals. However, in this study, in order to diminish the consequences of cell wall compensatory mechanisms that might alter the caspofungin-derived phenotypes [50,51,52], microscopy fluorescence imaging was performed during the first 3 h of treatment with both sublethal (non-inhibitory) and lethal (inhibitory) concentrations of caspofungin. Surprisingly, we observed a fungicidal effect and cell death at sublethal concentrations and a fungistatic effect of cell growth arrest and an absence of cell death at lethal concentrations. This fungistatic effect was transient, causing the expected morphological and lethal effect in the long term. Our study of the *GFP-bgs1^+^*, *GFP-bgs3^+^*, *GFP-bgs4^+^*, and *ags1^+^-GFP* (*GFP-GSs*) strains confirmed that caspofungin differentially affects cytokinesis progression and cell integrity, finding that lethal concentrations of the drug caused the following: (i) a decrease in the levels of GFP-Bgs1 fluorescence but not the expected decrease of the caspofungin-sensitive GFP-Bgs4 GS; (ii) altered locations of Bgs3, Bgs4, and Ags1; and (iii) a concomitant high accumulation of cells with CW-stained incomplete septa, whose septum synthesis was arrested or delayed with respect to plasma membrane ingression, resulting in CW-incomplete but GFP-complete septa. Finally, we found that the formation of CW-incomplete septa depended on the Pmk1 kinase of the cell wall integrity MAPK pathway.

## 2. Results and Discussion

### 2.1. Susceptibilities of WT, GFP-Tagged GSs, and pmk1∆ Strains to Caspofungin

The susceptibility of yeasts to caspofungin varies with the number of inoculated cells. Thus, greater cell numbers need higher drug concentrations to inhibit yeast growth [41,50]. Because proper imaging and preparation of yeast cells for fluorescence microscopy require cultures containing a high density of cells, we compared the caspofungin susceptibility of the WT, *GFP-GSs* (*GFP-bgs1^+^*, *GFP-bgs3^+^*, *GFP-bgs4^+^*, and *ags1^+^-GFP*) and *pmk1*∆ strains growing in microcultures inoculated with a high cell density (3 × 10^6^ cells/mL) that were later used in the fluorescence microscopy experiments (Table 1). In our experiments, we used the standard, optimal, and proper YES culture medium for both growth and subcellular location analyses in the fission yeast *S. pombe*. The minimal inhibitory concentration (MIC) for all the strains at a high cell density was in the range of 10 µg/mL of caspofungin (Table 1). Interestingly, the strain containing GFP-Bgs4 was slightly more susceptible to caspofungin, with its growth being largely compromised in the presence of 4 µg/mL of caspofungin (Table 1). All the *GFP-GS* strains exhibited the same morphology and growth rates as the untagged WT strain [21,22,25,31,33], suggesting that the corresponding chimeric protein was fully or almost completely functional. However, the possibility that tagging with GFP slightly alters the activity and/or the secondary conformation of the GSs cannot be ruled out. For example, a version of GFP-Bgs1 was shown to partially rescue the cytokinetic phenotype of cells lacking the paxillin Pxl1 [21,22,25,31,33]. In addition, since the major Bgs4 appears to be the most sensitive Bgs protein to echinocandins [50], it is possible that its GFP-tagging affects the activity to a greater extent than other GFP-Bgs proteins in cells growing in the presence of caspofungin.

**Figure 1:**
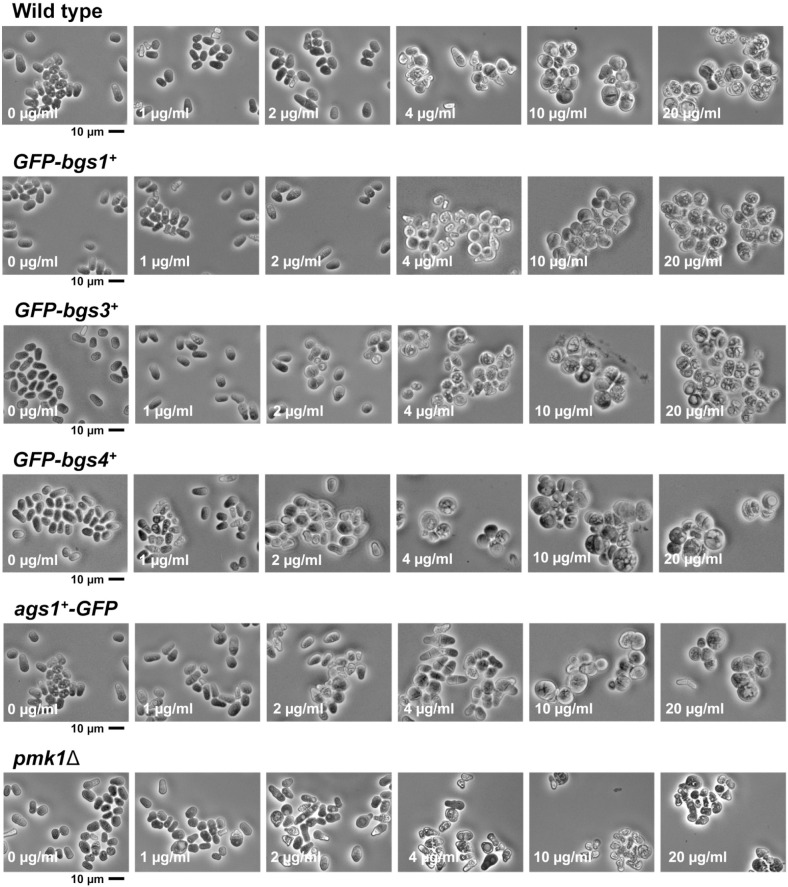
Morphology of WT, *GFP-GSs* and *pmk1*∆ cells after 24 h of growth in the presence of increasing doses of the echinocandin drug caspofungin. Early logarithmic phase cells of the indicated strains growing in YES (a specific culture medium for *S. pombe*) liquid medium at 28 °C were diluted to a high cell density of 3 × 10^6^ in microcultures of YES liquid medium containing either DMSO (0.4%, control) or increasing doses (1, 2, 4, 10, and 20 µg/mL) of the antifungal, grown with shaking for 24 h and imaged using phase-contrast microscopy. Scale bars: 10 µm.

On the basis of the obtained MICs, sublethal (non-inhibitory) concentrations of 2 and 4 µg/mL and lethal (inhibitory) concentrations of 10 and 20 µg/mL of caspofungin were chosen to analyze cytokinesis progression and GS localizations (see the following sections). Thereafter, starting at a high cell density, those microcultures were used to examine the cell morphology using phase-contrast microscopy after 24 h of growth in the presence of increasing concentrations of caspofungin. In all strains, caspofungin led to cells becoming aggregated, rounded, and swollen. The phenotype of rounded and swollen cells suggests a specific effect on Bgs4. This is in agreement with a similar spherical phenotype that is observed in *pbr1-6*, *cwg1-1*, and *orb11-59* mutant alleles of Bgs4 but that is absent in the *cps1-12*, *cps1-191*, and *cps1-N12* mutant alleles of Bgs1, which are elongated and multiseptated [23,53,54,55,56,57]. In most strains, this phenotype was first observed in cells treated with the sublethal concentration of 4 µg/mL of caspofungin (Figure 1). However, as regards the susceptibility assay (Table 1), the strain containing GFP-Bgs4 was slightly more susceptible, i.e., it already exhibited this phenotype in the presence of 2 µg/mL of caspofungin (Figure 1). Despite the *pmk1*∆ susceptibility to caspofungin being similar to that seen in other strains, after 24 h of treatment, the *pmk1*∆ cells seemed to be more affected by the drug, appearing darker, less healthy, and with more lysis than the WT and *GFP-GS* strains in the presence of the same concentrations of the drug (Figure 1). In conclusion, although the presence of GFP-Bgs4 seemed to slightly increase cell susceptibility to the drug, all these results showed that the WT, *GFP-GSs* and *pmk1*∆ strains exhibited the same range of susceptibility to caspofungin, with the MIC of 10 µg/mL.

### 2.2. Effect of Caspofungin in the Cellular Localization of the GSs

**Figure 2:**
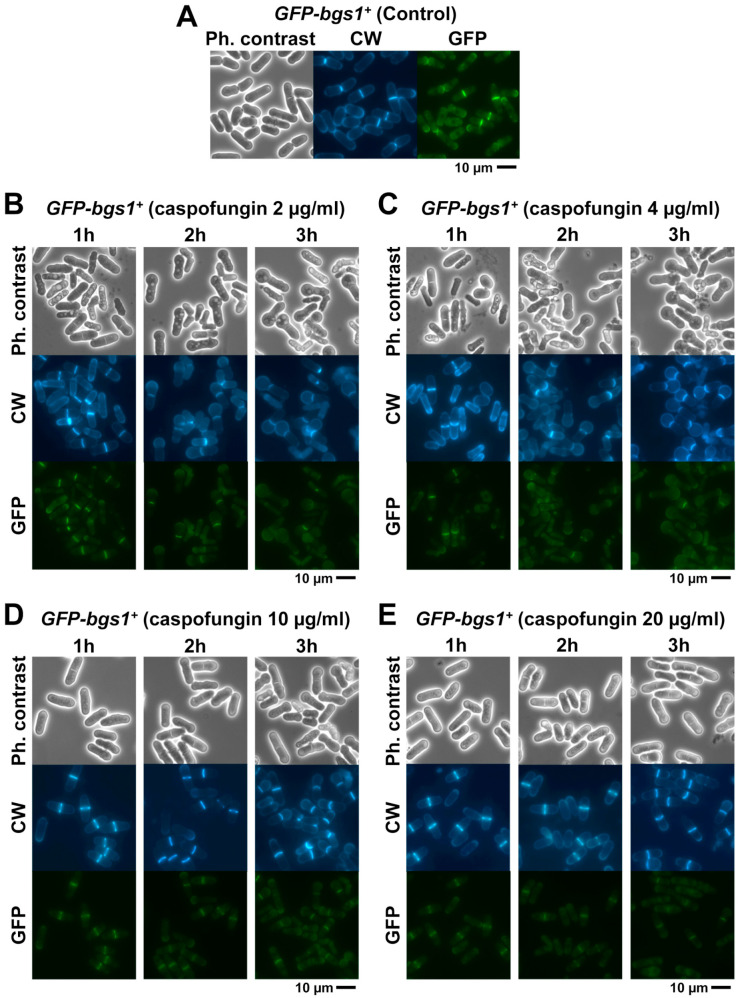
Cell morphology and GFP-Bgs1 localization in cells growing in the presence of sublethal and lethal concentrations of caspofungin. Phase-contrast and fluorescence micrographs of calcofluor white (CW)-stained *GFP-bgs1^+^* cells. Cells were grown to early log phase at 28 °C in YES liquid medium and imaged at the indicated times using phase-contrast and CW and GFP fluorescence microscopy either in the absence ((**A**): control) or in the presence of sublethal ((**B**): 2 µg/mL; (**C**): 4 µg/mL) or lethal ((**D**): 10 µg/mL; (**E**): 20 µg/mL) doses of the antifungal. Scale bars: 10 µm.

**Figure 3:**
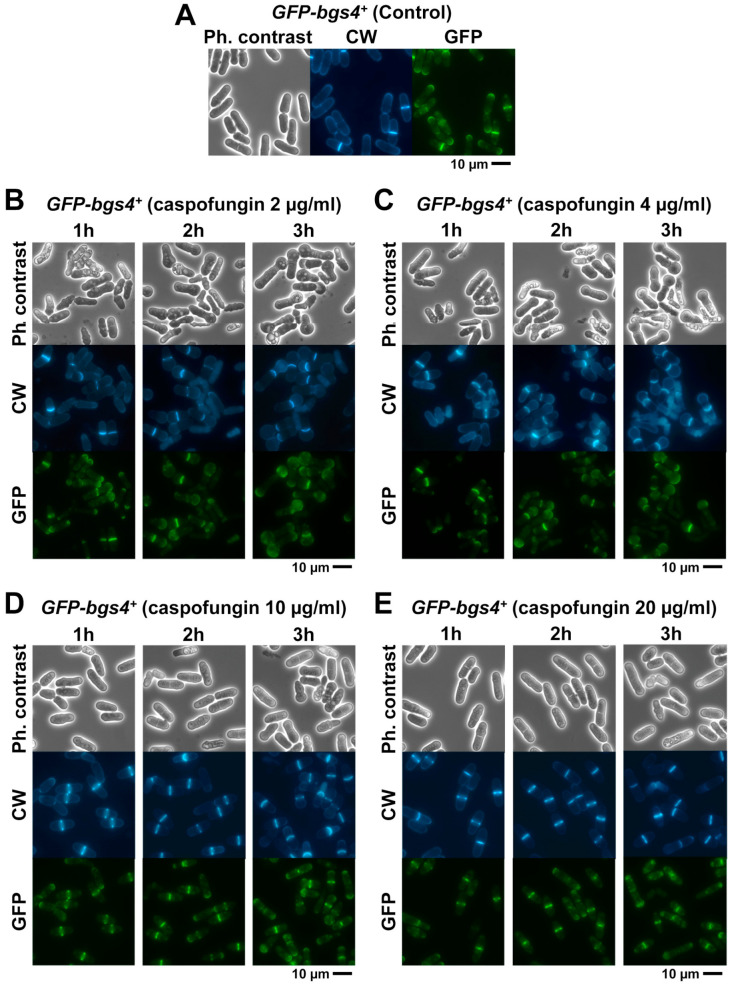
Cell morphology and GFP-Bgs4 localization in cells growing in the presence of sublethal and lethal concentrations of caspofungin. Phase-contrast and fluorescence micrographs of CW-stained *GFP-bgs4^+^* cells. Cells were grown as in Figure 2 and imaged at the indicated times using phase-contrast and CW and GFP fluorescence microscopy either in the absence ((**A**): control) or in the presence of sublethal ((**B**): 2 µg/mL; (**C**): 4 µg/mL) or lethal ((**D**): 10 µg/mL; (**E**): 20 µg/mL) doses of the antifungal. Scale bars: 10 µm.

**Figure 4:**
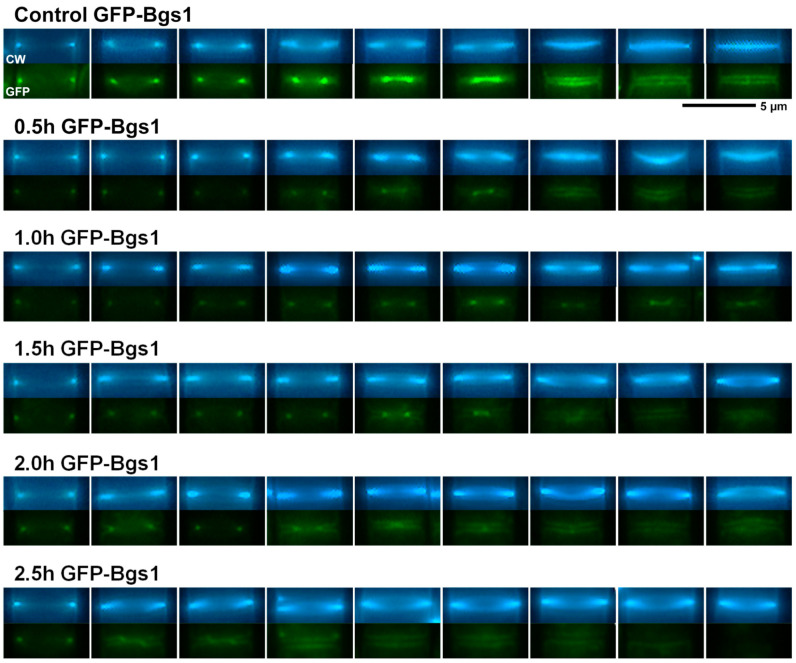
GFP-Bgs1 localization in the septum area in the absence or the presence of lethal doses of caspofungin. Cells were grown and imaged at the indicated times using CW and GFP fluorescence microscopy as in Figure 2 either in the absence (control) or in the presence of 20 µg/mL of the antifungal. Micrographs are magnifications of the septum area of representative cells in different stages of septum progression. Scale bars: 5 µm.

**Figure 5:**
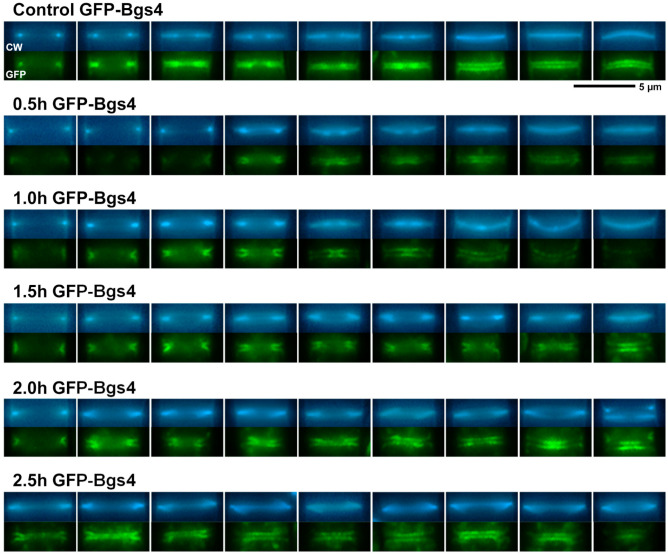
GFP-Bgs4 localization in the septum area in the absence or the presence of lethal doses of caspofungin. Cells were grown and imaged at the indicated times using CW and GFP fluorescence microscopy as in Figure 3 either in the absence (control) or in the presence of 20 µg/mL of the antifungal. Micrographs are magnifications of the septum area of representative cells in different stages of septum progression. Scale bars: 5 µm.

To analyze the effect of caspofungin on both the location of the GSs and the cell morphology, the strains containing Bgs1, Bgs3, Bgs4, and Ags1 tagged with GFP (*GFP-GSs*) were treated for 3 h with both sublethal (2 and 4 µg/mL) and lethal concentrations (10 and 20 µg/mL) of the echinocandin drug caspofungin. Samples were imaged every 30 min using phase-contrast and GFP and CW fluorescence microscopy. The localization and fluorescence of each GS subunit was compared with the localization of cells grown in the absence of the drug (control) (Figure 2, Figure 3 and Figures S1 and S2). Although the GS activity is related to the localization of the enzyme, the inhibition of a GS by caspofungin and modification of its localization are two different processes. Therefore, it is possible that GS localization is not affected while enzyme activity is inhibited. However, in the opposite case, there is a perfect correlation: if GS localization is reduced or absent, then unequivocally enzyme activity is affected or absent, at least in terms of exerting the correct function in the right place. In all cases, the caspofungin treatment induced a reduction in the fluorescence levels of the GSs in the cell, which was much more evident in the case of GFP-Bgs1 (Figure 2, Figure 3 and Figures S1 and S2). In *S. pombe*, echinocandins are thought to primarily inhibit βGS activity due to Bgs4 [30,50]; however, the presence of both lethal and sublethal concentrations of caspofungin mainly affected the levels of Bgs1 fluorescence, which were greatly reduced. Interestingly, Bgs1 location in the septum tip, which is associated with the contractile actomyosin ring, remained unchanged (Figure 4). The fluorescence of the other GSs, i.e., Bgs3, Bgs4, and Ags1, in the presence of lethal concentrations of caspofungin was only slightly reduced during the early times of treatment (Figure 5 and Figures S3 and S4). Contrary to what was observed in the reduced but unaltered Bgs1 localization, the treatment with lethal concentrations of caspofungin altered the location of Bgs4, Bgs3, and Ags1, whose fluorescence spread along the membrane that covers the septum (Figure 5 and Figures S3 and S4). Considering that Bgs1 may be important for restricting the localization of the rest of the GSs in the septal tip [21,25], the spreading of Bgs3, Bgs4, and Ags1 could be due to the reduced levels of Bgs1 in the septum tips in the presence of lethal concentrations of caspofungin. This hypothesis is also reinforced by the fact that Ags1 localization, which, theoretically, is not a target for caspofungin, is also altered in the presence of inhibitory concentrations of the drug. These results suggest that, in addition to Bgs4, lethal doses of caspofungin also affect the function and localization of Bgs1. Additionally, lethal concentrations of caspofungin might alter the function and/or localization of other components of the cytokinetic machinery and, thus, the decreased Bgs1 localization could be an indirect effect of this. A deep morphological analysis of the budding yeast *Saccharomyces cerevisiae* suggests that in addition to the β(1-3)glucan synthesis, echinocandin B also alters other functions of the multifunctional βGS Fks1, which is in accordance with the aforementioned phenomena [58].

### 2.3. Analysis of the Different Phenotypes Induced by Caspofungin during Early Times of Treatment

In addition to the localization of GSs, the *GFP-GS* strains were used to examine the cellular and septation phenotypes caused by the short exposure times (up to 3 h) with caspofungin. When examining the phenotype of cells in the presence of sublethal concentrations (2 and 4 µg/mL) of caspofungin for 3 h, it was seen that the drug caused lysis and death in all *GFP-GS* strains, with many of the cells appearing swollen at one or both of their poles throughout the treatment (Figure 2, Figure 3 and Figures S1 and S2). The rod-shaped yeast *S. pombe* grows at one (monopolar) or both poles (bipolar) during interphase [59]. Because most cells are monopolar, it was uncommon to detect swollen cells at both poles. This swelling results from the cell wall of the growing pole becoming weak and swollen because it does not incorporate new β(1-3)glucan chains in the presence of caspofungin. Thus, its structure is unable to counteract the internal turgor pressure of the cell pushing against the plasma membrane underneath the cell wall. As regards the MIC and cell morphology after 24 h of treatment with sublethal doses of the drug (Table 1 and Figure 1), the *GFP-bgs4^+^* strain was slightly more susceptible to the treatment with 2 µg/mL of caspofungin than the *GFP-bgs1^+^* strain. Thus, it exhibited slightly higher percentages of dead cells during early times of treatment. As explained above, Bgs4 is responsible for the majority of βGS activity and cell wall β(1-3)glucan detected in *S. pombe* and for its cell integrity. It is also the Bgs protein that is most sensitive to echinocandins [50]. Thus, it is possible that GFP tagging affects its activity to a greater extent than the other GFP-Bgs proteins in cells growing in the presence of caspofungin. Alternatively, GFP tagging might affect similarly to Bgs1, Bgs3, Bgs4, and Ags1. Thus, the difference in the percentages of lysed cells in each strain might be a consequence of the differential role of each synthase in cell integrity, with the absence of Bgs4 mainly causing death by cell lysis and cytoplasm release and the absence of Bgs1 impairing cytokinesis without causing cell lysis [20,32].

**Figure 6:**
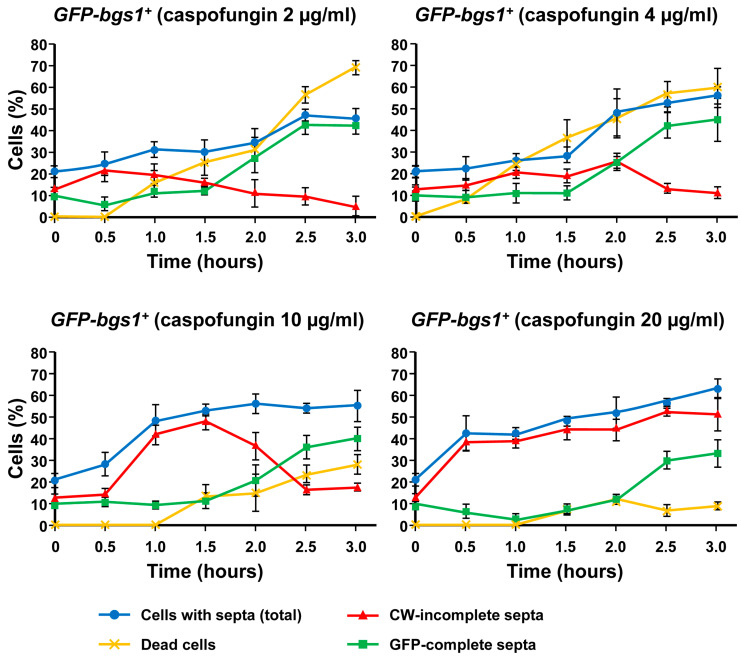
Percentages of the total *GFP-bgs1^+^* dead cells (yellow line), CW-stained total septa (blue line), CW-incomplete septa (red line), and GFP-complete septa (green line) either in the absence (time 0 h) or in the presence of sublethal (2 and 4 µg/mL) and lethal (10 and 20 µg/mL) concentrations of caspofungin. Cells were grown and imaged at the indicated times and drug concentrations as in Figure 2. Error bars indicate standard deviation.

**Figure 7:**
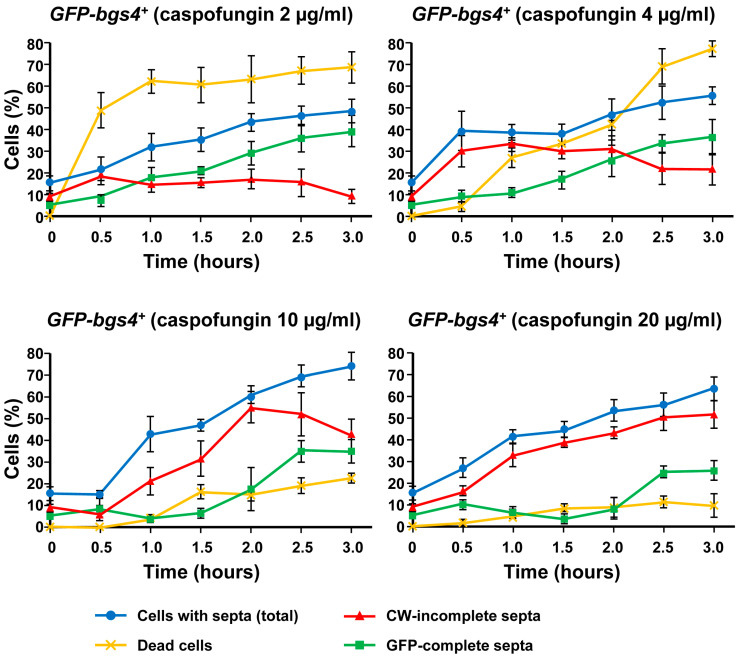
Percentages of the total *GFP-bgs4^+^* dead cells (yellow line), CW-stained total septa (blue line), CW-incomplete septa (red line), and GFP-complete septa (green line) either in the absence (time 0 h) or in the presence of sublethal (2 and 4 µg/mL) and lethal (10 and 20 µg/mL) concentrations of caspofungin. Cells were grown and imaged at the indicated times and drug concentrations as in Figure 3. Error bars indicate standard deviation.

In contrast, the phenotypes of cell death and pole swelling in the presence of lethal concentrations (10 and 20 µg/mL) of caspofungin were considerably reduced (Figure 2, Figure 3 and Figures S1 and S2). The differences in the percentage of cell death depending on the caspofungin concentration were confirmed by counting the total number and percentage of dead cells observed in the captured micrographs (yellow lines in Figure 6 and Figure 7; see Materials and Methods, Section 4). Despite the general reduction in cell death with lethal concentrations of caspofungin, most of the strains exhibited an intermediate phenotype of gradual decrease in dead cells in the presence of 4 and 10 µg/mL, while the cell death phenotype was largely suppressed in the presence of 20 µg/mL. Because for cell lysis to occur, the cells must be growing and actively modifying the structure of the cell wall and the septum, these results and those observed in the susceptibility assays (Table 1) indicate that the early treatment with sublethal doses of caspofungin does not arrest cell growth, resulting in a weakening of the cell wall in the areas of active remodeling and, thus, causing cell lysis in the cell middle, at the start of cell separation (lateral cell wall and septum dissolution), or in the growing regions at the poles [31]. However, the early treatment with lethal concentrations of caspofungin, most notably the 20 µg/mL dose, largely arrested cell growth and septation (see below). Thus, sublethal concentrations of caspofungin induced an initial fungicidal effect, which, over time, was attenuated and allowed the growth and division of those cells that survived this initial treatment (Figure 1). In contrast, lethal concentrations, most notably the 20 µg/mL dose, appeared to cause an initial fungistatic effect. However, these arrested cells eventually became very sick, aggregated, rounded, and swollen after 24 h in the presence of the drug (Figure 1) and eventually died in the longer term as was expected in the presence of a fungicidal drug. It is also important to consider that the effect of inducing a spherical phenotype is similar to that observed in some mutants of Bgs4, suggesting a direct effect on Bgs4 [53,54,55]. In addition, these results are in accordance with those of previous reports using untagged WT and *pbr1-8* strains [30,50].

### 2.4. Lethal Concentrations of Caspofungin Induce Uncoupling of Delayed Septum Synthesis and Advanced Plasma Membrane Ingression

As mentioned above, during cytokinesis, the GSs are responsible for the synthesis of the different wall structures of the division septum [8,19]. Therefore, in parallel with the analysis of the localization of the GSs and cell phenotypes in the presence of caspofungin (Figure 2, Figure 3 and Figures S1 and S2), CW and GFP-GS fluorescence of the captured micrographs were used to examine the septation by quantifying the percentages of cells with open (CW-incomplete), closed (GFP-complete), and total septa throughout the 3 h of treatment with both non-inhibitory and inhibitory concentrations of caspofungin (red, green, and blue lines in the graphs in Figure 6 and Figure 7; see Materials and Methods, Section 4). In general, the treatment with both sublethal and lethal doses of caspofungin caused an increase in the population of cells displaying septa in all the analyzed *GFP-GS* strains. After 3 h, these percentages varied from 40–50% of the cells in the presence of sublethal concentrations to 60–70% in the presence of inhibitory concentrations (blue lines in the graphs in Figure 6 and Figure 7). Thereafter, the percentages of cells exhibiting CW-stained incomplete septa were examined (red lines in Figure 6 and Figure 7). In the presence of sublethal concentrations of the drug, the majority of cells exhibited CW-complete septa and, thus, the percentages of CW-incomplete septa decreased over the 3 h of treatment. Contrarily, lethal concentrations of the drug induced an accumulation of arrested cells that exhibited an increase in CW-incomplete septa. However, while the treatment with 10 µg/mL induced a peak of cells with CW-incomplete septa between 1.5 and 2 h, which is in agreement with the intermediate phenotype of cell death, the 20 µg/mL treatment induced a higher accumulation of cells with CW-incomplete septa, which was maintained and increased during at least the 3 h of treatment (red lines in Figure 6 and Figure 7). Finally, the percentages of cells exhibiting a closed septum, as observed using GFP-GS fluorescence in the septum membrane, were also quantified (GFP-complete septa, green lines in Figure 6 and Figure 7). In the presence of sublethal concentrations of the drug, there was a perfect correlation between the decrease in CW-incomplete septa (red line) and the increase in GFP-complete septa (green line). Thus, the majority of cells exhibited complete septa according to both CW and GFP-GS fluorescence. Interestingly, the treatment with lethal concentrations of caspofungin (most notably after 1.5 to 2 h of treatment with 20 µg/mL) showed that most of the CW-incomplete septa were complete according to GFP-GS fluorescence, indicating that lethal concentrations of caspofungin induce uncoupling of delayed CW staining (synthesis of the primary septum) and advanced ingression of the plasma membrane (green lines in Figure 6 and Figure 7). This phenotype was confirmed by magnifying the septum region of cells treated with 20 µg/mL of caspofungin (Figure 4, Figure 5 and Figures S3 and S4). Thus, after 1.5 h of treatment with caspofungin, many CW-incomplete septa appeared complete according to the localization of the GFP-GSs, with the two plasma membranes surrounding the septum appearing as two continuous and completely separated structures (Figure 4, Figure 5 and Figures S3 and S4). In summary, we found that treatment with lethal doses of caspofungin, in particular 20 µg/mL, altered cytokinesis progression. Early in treatment, the cells exhibited an accumulation of open septa, as noted via CW-staining or plasma membrane observation. This phenotype of slowed septum progression has also been described in cells affected in the function of Bgs1, which is the GS responsible for the synthesis of the primary septum, and its function is required for septum progression [23,25,57]. Later, a separation between slower primary septum synthesis and faster plasma membrane ingression was observed. The absence of Bgs4-synthesized β(1-3)glucan was also described to cause both delayed primary septum progression and its uncoupling from fast membrane ingression [25,31]. Globally, these results suggest that lethal doses of caspofungin affect the function of at least Bgs1 and Bgs4. Additionally, the diminished localization of Bgs1 in the septum caused by lethal doses of caspofungin might also have affected the location and function of Bgs3, Bgs4, and Ags1 (see above) and, thus, the steady progression of the cleavage furrow during septation.

### 2.5. The Absence of Pmk1 Suppresses the Phenotypes Caused by Short Treatments with Sublethal and Lethal Doses of Caspofungin

**Figure 8:**
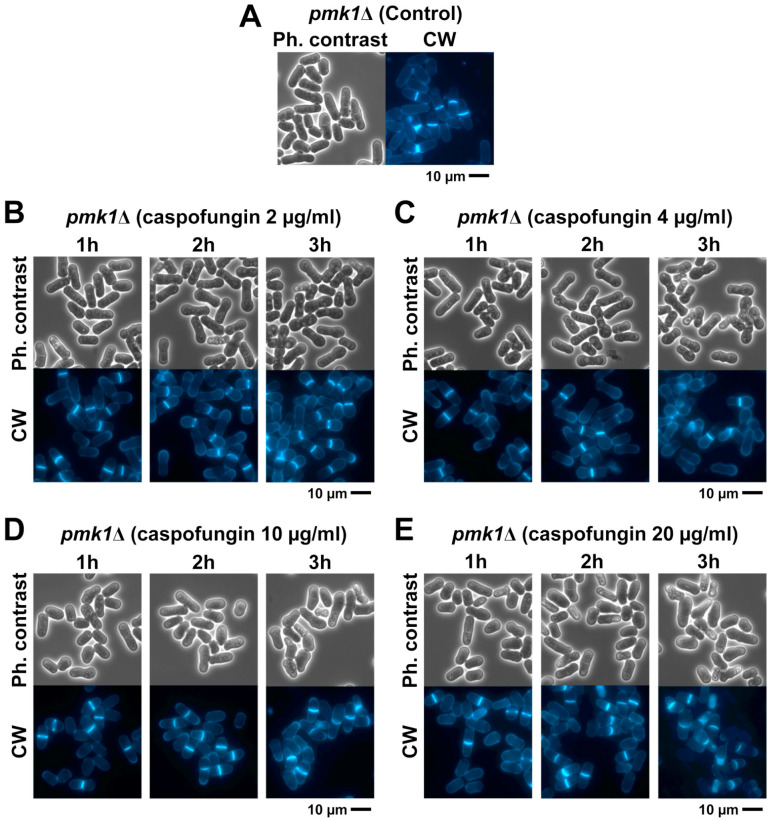
Cell morphology and septum formation of *pmk1*∆ cells growing in the presence of sublethal and lethal concentrations of caspofungin. Phase-contrast and fluorescence micrographs of CW-stained *pmk1*∆ cells. Cells were grown to early log phase at 28 °C in YES liquid medium and imaged at the indicated times using phase-contrast and CW fluorescence microscopy either in the absence ((**A**): control) or in the presence of sublethal ((**B**): 2 µg/mL; (**C**): 4 µg/mL) or lethal ((**D**): 10 µg/mL; (**E**): 20 µg/mL) doses of the antifungal. Scale bars: 10 µm.

**Figure 9:**
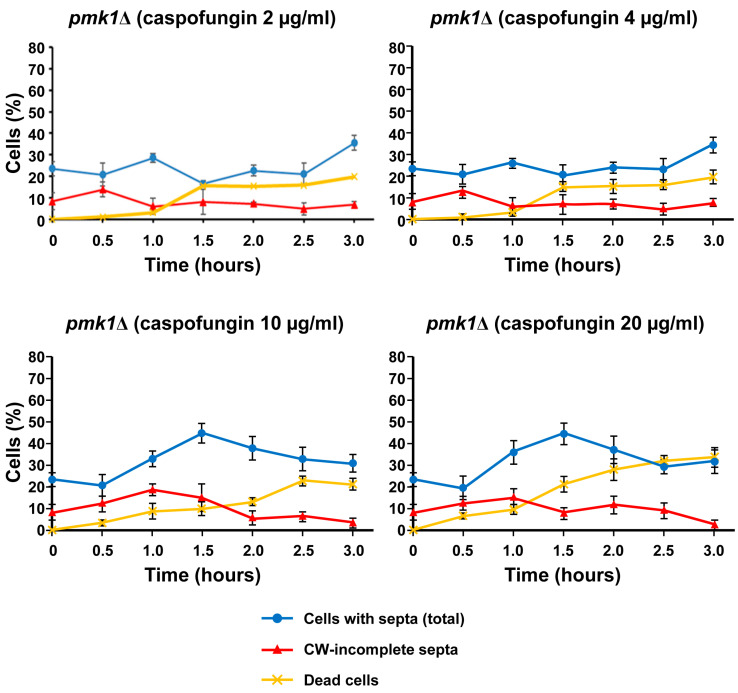
Percentages of the total *pmk1*∆ dead cells (yellow line), CW-stained total septa (blue line), and CW-incomplete septa (red line) either in the absence (time 0 h) or in the presence of sublethal (2 and 4 µg/mL) and lethal (10 and 20 µg/mL) concentrations of caspofungin. Cells were grown and imaged at the indicated times and drug concentrations as in Figure 8. Error bars indicate standard deviation.

In the fission yeast, the mitogen-activated protein kinase (MAPK) cell integrity pathway is important in multiple functional aspects of the life cycle, either during unperturbed growth or in response to external changes that cause cellular stress, such as damage to the cell wall and osmotic changes [60]. The final effector of the cell integrity pathway is the MAPK Pmk1, which becomes active in response to cellular stress. Pmk1 is also active during cytokinesis and has been implicated in the maintenance of cell wall stiffness surrounding the growing poles [60,61]. Thus, we speculated that the accumulation of CW-incomplete septa in cells treated with lethal doses of caspofungin could be a delay to allow the activation of the cell wall damage-repair mechanism, which is dependent on the activation of the cell integrity pathway. As with the *GFP-GS* strains, the *pmk1*∆ strain was grown in the presence of both sublethal and lethal concentrations of caspofungin and imaged every 30 min over 3 h for both phase-contrast imaging and CW-staining fluorescence (Figure 8). Thereafter, we quantified the percentage of cell death in the captured micrographs. In contrast to what was observed with the *GFP-GS* strains, the percentages of cell death in the presence of sublethal concentrations of caspofungin (2 and 4 µg/mL) were reduced throughout the entire treatment. Thus, the *pmk1*∆ strain reached only 20% cell death after 3 h of the sublethal treatment (yellow lines in the graphs in Figure 9), while the strains containing GFP-Bgs1 and GFP-Bgs4 exhibited 60–70% cell death (yellow lines Figure 6 and Figure 7). Similarly, the opposite effect was observed in the treatment with a 20 µg/mL lethal dose. The percentage of cell death was almost 40% in the *pmk1*∆ strain (yellow line in the graphs in Figure 9), while the percentages of death were only 10% in the cells containing GFP-Bgs1 and GFP-Bgs4 (yellow lines in the graphs in Figure 6 and Figure 7). In the case of the intermediate phenotypes induced in the presence of 10 µg/mL of the drug, the percentages of cell death were quite similar between the analyzed strains, with the *pmk1*∆ strain exhibiting 20% cell death and the *GFP-bgs1^+^* and *GFP-bgs4^+^* strains exhibiting 30% and 25%, respectively. When the septation was examined, we observed that sublethal doses of the drug (2 and 4 µg/mL) did not increase the percentages of *pmk1*∆ cells exhibiting CW-stained total septa (blue lines in Figure 9). In the case of the treatment with lethal doses (10 and 20 µg/mL), the percentage peaked at 45% after 1.5 h of treatment and then decreased to 30% (blue lines in Figure 9). Similarly, treatment of the *pmk1*∆ strain with both sublethal and lethal concentrations of caspofungin did not cause the accumulation of cells with CW-incomplete septa that was observed in the *GFP-GS* strains treated with lethal concentrations of the drug (red lines in the graphs in Figure 9). Globally, these results suggest that Pmk1 might be responsible for the slowed septum progression observed in the presence of lethal concentrations of caspofungin. Although Pmk1 is constitutively localized in both the cytoplasm and nucleus, it has also been detected in the mitotic spindle and in the septum during cytokinesis, which is in agreement with the aforementioned results. Interestingly, it has been proposed that the cell integrity pathway is important for delaying cytokinesis progression through the activation of Pck2 and Pmk1 by the Rgf1–Rho1 complex [62]. In addition, the absence of Pmk1 also suppressed the cell death phenotypes caused by treatment with sublethal doses of caspofungin (2 and 4 µg/mL, yellow lines in Figure 9). Since the majority of cell death occurs once the septum is completed at the start of cell separation as a result of cell wall degradation [31,50], Pmk1 (or the cell wall integrity pathway) could inhibit or delay cell wall and septum dissolution in the presence of cell wall damage. In fact, cell integrity pathway mutants lacking Mkh1, Pek1, and Pmk1 or Pmk1 hyperactivation also lead to defects in cell separation [60,63,64].

## 3. Concluding Remarks

We previously studied the differential effect of echinocandins in cytokinesis progression and cell integrity during early times of treatment [50]. Herein, we began to analyze the septal and tip localization of the four conserved and essential fungal GSs from the fission yeast (Bgs1, Bgs3, Bgs4, and Ags1) during the early times (up 3 h) of treatment with both lethal and sublethal doses of the echinocandin drug caspofungin. To the best of our knowledge, this is the first study to analyze the subcellular localization of the catalytic subunits of βGS and αGS in the plasma membrane of both the septum and cell ends during early times of treatment with caspofungin.

Although a previous study described an initial delocalization from the cell tips to vacuoles of *Aspergillus fumigatus* βGS Fks1 in the presence of both growth-inhibitory (0.5 µg/mL) and paradoxical growth-inducing (4 µg/mL) doses of caspofungin after the long times of 24 and 48 h of treatment, and without examining the Fks1 location in the septum and the septum formation [65], it is difficult to compare their results with those described here, as ours are focused on the early treatment period with caspofungin. Previous studies suggested that the major βGS Bgs4 from *S. pombe* appears to be the main target for cell wall antifungals [30,50]. However, herein, we found that lethal doses of caspofungin during the early times of treatment induced a decrease in the levels of GFP-Bgs1 fluorescence that was accompanied by both an altered localization of Bgs3, Bgs4, and Ags1 and the concomitant accumulation of cells with CW-stained incomplete septa that, in fact, were complete when observed with the integral plasma membrane GFP-GSs. This demonstrates that septum synthesis was delayed with respect to plasma membrane ingression. These results are in accordance with those previously reported concerning uncoupled septation with delayed septum synthesis in the absence of Bgs4 [31]. The uncoupling of delayed septum synthesis and advanced plasma membrane ingression observed in either cells treated with lethal doses of the drug or cells depleted of Bgs4 confirms that Bgs4 is the main target for caspofungin in fission yeast. Furthermore, our results showing the selective and specific delocalization of GFP-Bgs1 during the early times in the presence of caspofungin suggest that not only Bgs4 but also Bgs1 is a target for caspofungin. Further studies are required to explore whether this is an effect specific to caspofungin or whether this is general for other or all echinocandins.

Thus, the effect of echinocandins in the GSs of fission yeasts seems to be more complicated than previously believed. Despite Bgs1 having a minor role in the global synthesis of the cell wall β(1-3)glucan, its function is essential for cytokinesis and appears to also be important in controlling the localization of the rest of the GSs in the septum [21,25]. Therefore, the altered locations of Bgs3, Bgs4, and Ags1 in cells treated with lethal doses of caspofungin could be an indirect effect of the reduced levels of Bgs1 in the septum membrane. Interestingly, cell morphological quantitative analyses of the budding yeast *S. cerevisiae* suggest that echinocandin B impacts not only β(1-3)glucan synthesis but also other functions of the multifunctional βGS Fks1 [58]. In order to unravel how caspofungin specifically diminish the localization of contractile actomyosin ring associated Bgs1 in the septum, further experiments focused on analyzing the location and function of additional components of the cytokinetic machinery in the presence of caspofungin are required. Additionally, new studies of the localization of the GSs in cells treated with sublethal and lethal doses of other echinocandins (micafungin, anidulafungin, rezafungin, etc.) and other families of specific βGS antifungals (enfumafungin and papulacandins), during both short and long times of treatment, might help elucidate the mechanism of action and the specificity of the different cell wall antifungals that are currently available. Because the high conservation of β(1-3)glucan synthesis and the corresponding βGS enzymes observed throughout the fungi kingdom, the results obtained in this study are of interest for future studies concerning pathogenic fungi such as *Candida* sp. and *Aspergillus* sp.

## 4. Materials and Methods

### 4.1. Strains and Culture Conditions

The *S. pombe* strains examined in this study are listed in Table 2. Strains *GFP-bgs1^+^* (1722), *GFP-bgs3^+^* (3321), *GFP-bgs4^+^* (2364), and *ags1^+^-GFP* (3166) have been previously described [21,22,25,31,33] and contain the *bgs1*Δ::*ura4^+^*, *bgs3*Δ::*ura4^+^*, *bgs4*Δ::*ura4^+^*, and *ags1*Δ 3’UTR*ags1*^+^::*ags1_3704-7233_*:*ura4^+^* deletions and an integrated copy of SmaI-cut pJK-*GFP-12A-bgs1^+^*, PacI-cut pJK*GFP-12A-bgs3^+^*, StuI-cut pJK-*GFP-12A-bgs4^+^*, and AgeI-cut pJK-*ags1_1-6267_-12A-GFP-12A* (*leu1^+^* selection), which direct their integrations at the SmaI site of the *bgs1^+^* promoter sequence (nt-748) adjacent to *bgs1*Δ::*ura4^+^*, the PacI site of the *bgs3^+^* promoter sequence (nt-1857) adjacent to *bgs3*Δ::*ura4^+^*, the StuI site of the *bgs4^+^* promoter sequence (nt-1320) adjacent to *bgs4*Δ::*ura4+*, and the AgeI site of the *ags1^+^* coding sequence (nt +6025) in *ags1*Δ 3’UTR*ags1*^+^::*ags1_3704-7233_*:*ura4^+^*, respectively.

The standard rich yeast growth medium (YES from “Yeast Extract with Supplements”: Yeast Extract, Dextrose, and Supplements of Adenine, Histidine, Leucine, Uracil, and Lysine) has been described previously [66]. The standard YES medium for *S. pombe* cell growth was used for the determination of the sensitivity and MIC values of the tested caspofungin as described [30,40,50,53,54,56]. Cell growth was monitored by measuring the A_600_ of early log-phase cell populations in a SmartSpec 3000 spectrophotometer (Bio-Rad; Hercules, CA, USA; A_600_ 0.1 = 10^6^ cells/mL).

### 4.2. Antifungal Drugs and Susceptibility Assays

The caspofungin used in this study was a generous gift from Merck Sharp and Dohme. It was maintained at −20 °C in a stock solution (10 mg/mL in DMSO) and assayed at the final concentrations specified in the text, tables, and figures. For microculture assays of large numbers of samples, log-phase cultures grown in YES medium were diluted to a cell density of 3 × 10^6^ cells/mL in YES medium containing increasing concentrations of the echinocandin drug caspofungin (1, 2, 4, 10, 20, and 40 µg/mL) or an equal volume of solvent (0.4% DMSO), which was the control cell culture. The cell cultures were grown in an orbital roller at 28 °C, and turbidity was examined after 24 h of growth, affording values ranging from - (no turbidity, wild-type cells in the presence of a lethal concentration of antifungal) to ± (0–5% turbidity), + (5–10% turbidity), +± (10–15% turbidity), ++ (15–25% turbidity), ++± (25–37.5% turbidity), +++ (37.5–50% turbidity), +++± (50–62.5% turbidity), ++++ (62.5–75% turbidity), ++++± (75–87.5% turbidity), and +++++ (87.5–100%, total turbidity, wild-type cells in the absence of antifungal). The MIC value was determined as the minimal concentration of caspofungin that induced complete inhibition of cell growth (no growth or residual growth: - or +) after 24 h of growth in YES medium at 28 °C. The values were calculated from at least three independent experiments.

### 4.3. Microscopy Techniques and Data Analysis

Images of cells after 24 h of growth in the presence of caspofungin (Figure 1) were directly obtained from the microcultures used for the susceptibility assays with a Nikon Eclipse 50i microscope, a Nikon Plan FLUOR 20×/0.45 objective, a Nikon Ds-Fi1 digital camera, and a Nikon Digital Sight DS-L2 control unit, as previously described [50]. For cell wall staining and GFP-GS localization, early log-phase cells with or without GFP-labelled GS proteins were grown at 28 °C in YES liquid medium. Before imaging, the cells were concentrated (1000× *g*, 1 min) and resuspended by adding a solution of Calcofluor White (CW; 50 μg/mL final concentration; Fluorescent Brightener 28 Sigma-Aldrich, Burlington, MA, USA) to the sample and visualized using the appropriate filters.

Images were obtained with a Leica DM RXA fluorescence microscope, a PL APO 63×/1.32 oil PH3 objective, a digital camera (DFC350FX; Leica, Wetzlar, Germany), and CW4000 cytoFISH software (Leica). Images were processed with the Adobe Photoshop software. All the analyses were repeated in three independent experiments and representative images of the analyzed phenotypes were indistinctly selected from the experiments.

### 4.4. Quantification of Dead Cells and Septated Cells

The graphs in Figure 6 and Figure 7 show the percentages of dead cells (yellow lines), the percentages of cells with a septum (total septa, blue lines), the percentages of cells exhibiting a complete septum observed by the GFP-GS fluorescence associated to the septum membrane (GFP-complete septa, green lines), and the percentages of cells with an incomplete septum observed by CW staining (CW-incomplete septa, red lines). The graphs in Figure 9 show the percentages of dead cells, the percentages of cells with a septum (total), and the percentages of cells with an incomplete septum observed exclusively by CW staining (CW-incomplete septa).

In all graphs (Figure 6, Figure 7 and Figure 9), the percentages were calculated with respect to the total number of visualized cells. In the septum analyses, the cells in the cell separation process (when septum degradation had already started) were not considered to have a septum. Similarly, because cell death in the septum area always occurs during cell separation [31], the dead cells exhibiting a residual septum died during the cell separation process and, therefore, were not considered to contain a septum. The quantification of dead cells was considered the sum of the following: (i) cells with lysis and leakage of the cytoplasmic content in either the poles or the septum area; and (ii) cells with shrinkage and entry of the CW fluorochrome into the dead cell. In wild-type cells, the percentages of cells with a septum (total) were the sum of cells with complete and incomplete septa. Because the treatment with lethal doses of caspofungin induced an accumulation of cells with CW-stained incomplete septa (red lines) that, in fact, appeared complete when observed with the integral plasma membrane GFP-Bgs1 or GFP-Bgs4, the percentages of cells with complete septa were quantified with respect to the septum membrane through the GFP-Bgs1 and GFP-Bgs4 fluorescence (green lines). Thus, when CW staining and plasma membrane GFP were uncoupled, the majority of the cells with septa (total) exhibited an open septa according to CW staining despite the high percentage of cells with complete septa by GFP fluorescence (Figure 4, Figure 5 and Figures S3 and S4). As a result, the sum of CW-incomplete and GFP-complete septa is higher than the number of total septa. Contrarily, when CW staining and plasma membrane GFP were coupled, the sum of CW-incomplete and GFP-complete septa produced the exact value of total septa.

## Figures and Tables

**Table 1:** In vivo susceptibility assay to caspofungin of *S. pombe* wild-type strains with the Bgs1, Bgs3, Bgs4, and Ags1 glucan synthases fused to GFP and strain lacking the Pmk1 kinase of the cell integrity pathway.

Strains	Caspofungin (μg/mL)						
	0	1	2	4	10	20	40
WT	+++++	++++±	++++±	+++±	±	-	-
*GFP-bgs1^+^*	+++++	+++++	++++±	+++	-	-	-
*GFP-bgs3^+^*	+++++	+++++	++++	+++	-	-	-
*GFP-bgs4^+^*	+++++	++++	+++	+±	-	-	-
*ags1^+^-GFP*	+++++	++++±	+++±	+++	-	-	-
*pmk1*Δ	+++++	++++±	++++	++++	±	-	-

WT: Wild type. The minimal inhibitory concentration (MIC) value was determined as the minimal concentration for no growth or residual growth (-, ±, or +) after 24 h of growth in YES medium at 28 °C. Cells were diluted to a cell density of 3 × 10^6^ cells/mL and grown in microcultures of YES liquid medium with increasing concentrations (0, 1, 2, 4, 10, 20, and 40 μg/mL) of caspofungin. Values are the average of three to four independent experiments. The quantitative data of absorbance was simplified and grouped as follows: - indicates no growth (no turbidity), ± (0–5% turbidity), + (5–10% turbidity), +± (10–15% turbidity), ++ (15–25% turbidity), ++± (25–37.5% turbidity), +++ (37.5–50% turbidity), +++± (50–62.5% turbidity), ++++ (62.5–75% turbidity), ++++± (75–87.5% turbidity), and +++++ indicates total growth (87.5–100% turbidity).

**Table 2:** Fission yeast strains used in this study.

Strain	Genotype	Source
33	972 h	P. Munz ^a^
1722	*leu1-32 ura4-*Δ*18 his3-*Δ*1 bgs1*Δ*::ura4^+^* P*bgs1^+^::GFP-12A-bgs1^+^:leu1^+^* h^−^	J. Ribas
3321	*leu1-32 ura4-*Δ*18 his3-*Δ*1 bgs3*Δ*::ura4^+^* P*bgs3^+^::GFP-12A-bgs3^+^:leu1^+^* h^+^	J. Ribas
2364	*leu1-32 ura4-*Δ*18 his3-*Δ*1 bgs4*Δ*::ura4^+^* P*bgs4^+^::GFP-12A-bgs4^+^:leu1^+^* h^−^	J. Ribas
3166	*leu1-32 ura4-*Δ*18 his3-*Δ*1 ade6-M210 ags1*Δ 3’UTR*ags1^+^::ags1^+^-12A-GFP-12A:leu1^+^:ura4*^+^ h^−^	J. Ribas
6367	*leu1-32 ura4-*Δ*18 pmk1*Δ*::ura4^+^* h^+^	T. Toda ^b^

^a^ Institute of General Microbiology, University of Bern, Switzerland. ^b^ Department of Molecular Biotechnology, Hiroshima Research Center for Healthy Aging (HiHA), Graduate School of Advanced Sciences of Matter, Hiroshima University, Higashi-Hiroshima, Japan.

## Data Availability

Raw data are available from the corresponding author upon request.

## Supplementary Materials

The supporting information can be downloaded at: https://www.mdpi.com/article/10.3390/ijms24054299/s1.
