# Supplementary material for: Analysis of the Localization of Schizosaccharomyces pombe Glucan Synthases in the Presence of the Antifungal Agent Caspofungin
Source: Int J Mol Sci. 2023 Feb 21;24(5):4299. doi: 10.3390/ijms24054299 (PMC10002279; doi:10.3390/ijms24054299)
Supplement: Supplementary file 1 [file ijms-24-04299-s001.zip › ijms-2158817-supplementary.pdf]

Supplementary Material

Figure S1. Cell morphology and GFP-Bgs3 localization in cells growing in the presence of sublethal and lethal concentrations of caspofungin. Phase-contrast and fluorescence micrographs of calcofluor white (CW)-stained *GFP-bgs3*$^{+}$ cells. Cells were grown to early log-phase at 28ºC in YES medium and imaged at the indicated times for phase-contrast and for CW and GFP fluorescence either in the absence (A, control) or in the presence of sublethal (B, 2 µg/ml and C, 4 µg/ml) or lethal (D, 10 µg/ml and E, 20 µg/ml) doses of the antifungal. Scale bars, 10 µm.

Figure S2. Cell morphology and Ags1-GFP localization in cells growing in the presence of sublethal and lethal concentrations of caspofungin. Phase-contrast and fluorescence micrographs of CW-stained *ags1*$^{+}$*-GFP* cells. Cells were grown as in figure S1 and imaged at the indicated times for phase-contrast and for CW and GFP fluorescence either in the absence (A, control) or in the presence of sublethal (B, 2 µg/ml and C, 4 µg/ml) or lethal (D, 10 µg/ml and E, 20 µg/ml) doses of the antifungal. Scale bars, 10 µm.

Figure S3. GFP-Bgs3 localization in the septum area in the absence or the presence of lethal doses of caspofungin. Cells were grown and imaged at the indicated times for CW and GFP fluorescence as in figure S1 either in the absence (control) or the presence of 20 µg/ml of the antifungal. Micrographs are magnifications of the septum area of representative cells in different stages of septum progression. Scale bars, 5 µm.

Figure S4. Ags1-GFP localization in the septum area in the absence or the presence of lethal doses of caspofungin. Cells were grown and imaged at the indicated times for CW and GFP fluorescence as in figure S2 either in the absence (control) or the

presence of 20 μg/ml of the antifungal. Micrographs are magnifications of the septum area of representative cells in different stages of septum progression. Scale bars, 5 μm.

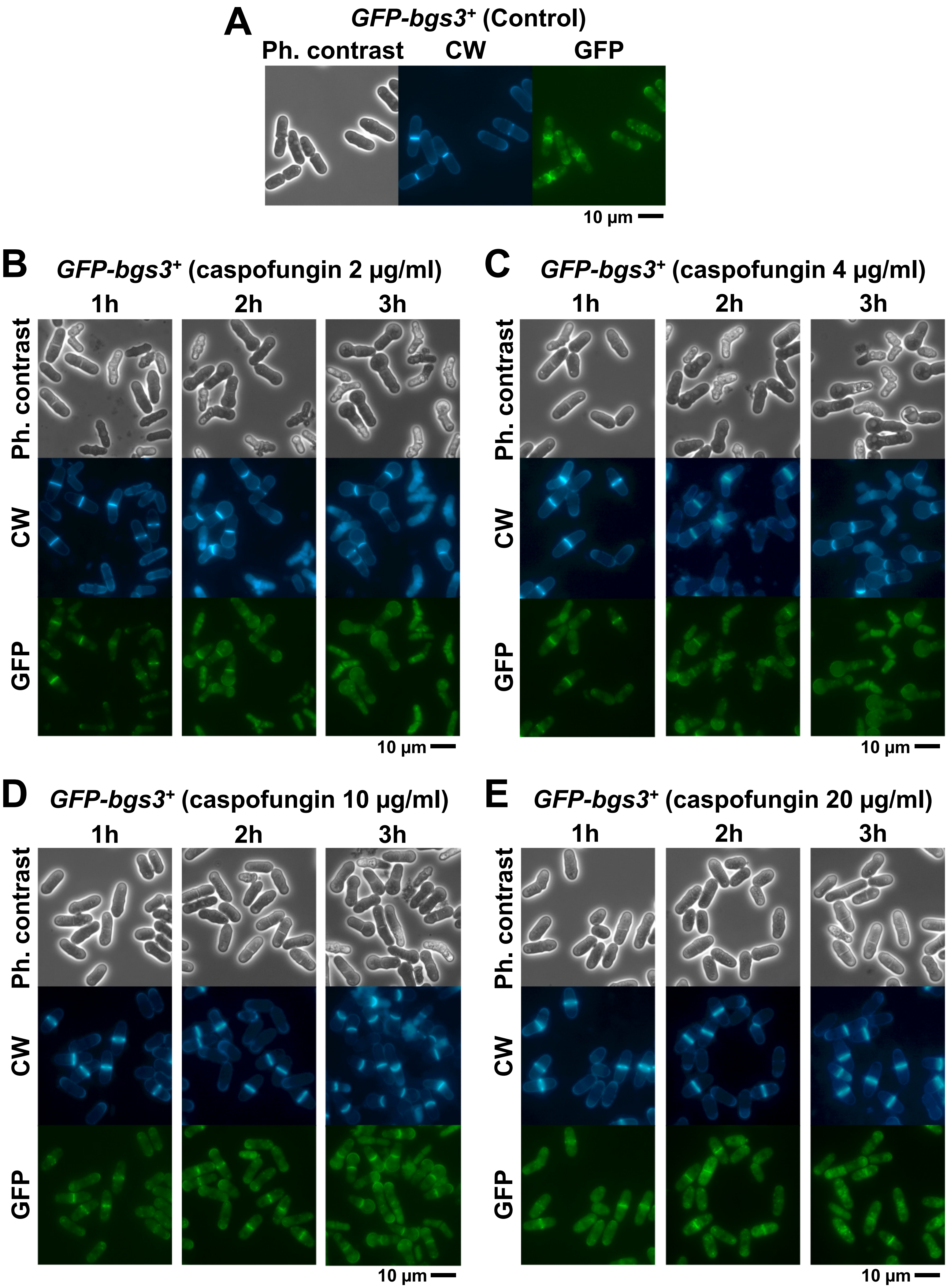
A
GFP-bgs3+ (Control)
Ph. contrast
CW
GFP
10 µm
B
GFP-bgs3+ (caspofungin 2 µg/ml)
1h
2h
3h
Ph. contrast
CW
GFP
10 µm
C
GFP-bgs3+ (caspofungin 4 µg/ml)
1h
2h
3h
Ph. contrast
CW
GFP
10 µm
D
GFP-bgs3+ (caspofungin 10 µg/ml)
1h
2h
3h
Ph. contrast
CW
GFP
10 µm
E
GFP-bgs3+ (caspofungin 20 µg/ml)
1h
2h
3h
Ph. contrast
CW
GFP
10 µm

Figure S1

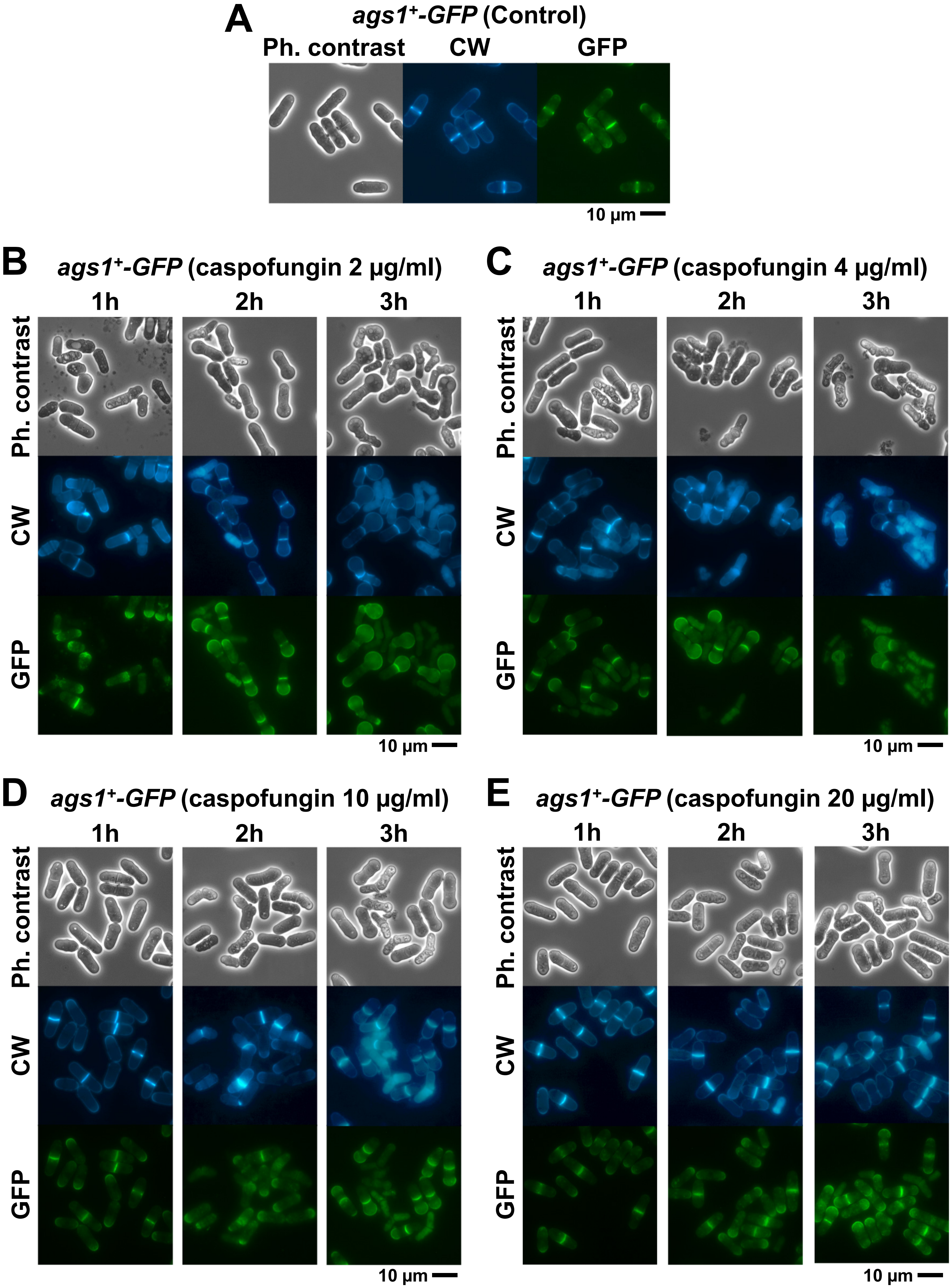
A
ags1+-GFP (Control)
Ph. contrast
CW
GFP
10 µm
B
ags1+-GFP (caspofungin 2 µg/ml)
C
ags1+-GFP (caspofungin 4 µg/ml)
D
ags1+-GFP (caspofungin 10 µg/ml)
E
ags1+-GFP (caspofungin 20 µg/ml)
1h
2h
3h
Ph. contrast
CW
GFP
10 µm

Figure S2

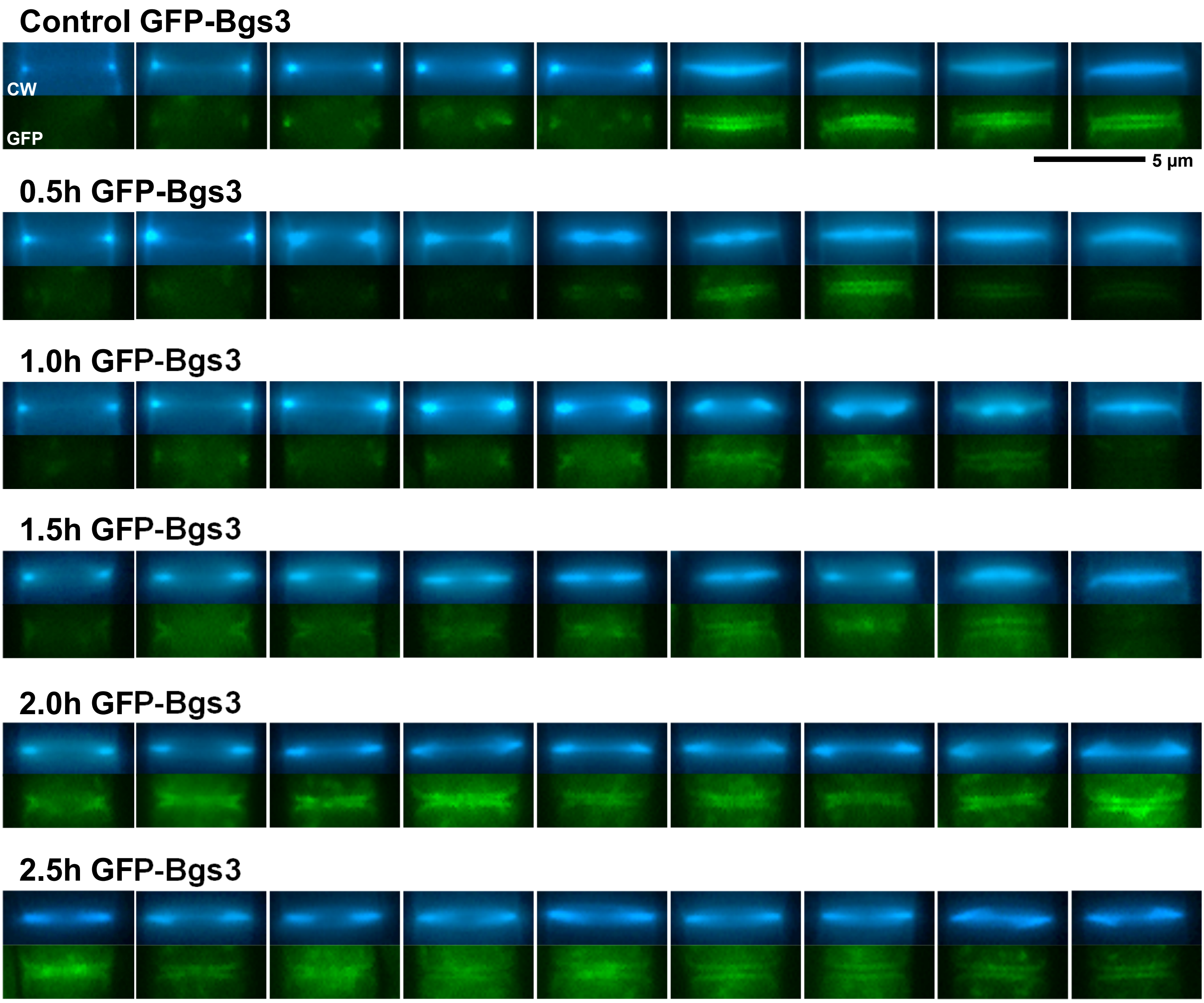
Control GFP-Bgs3
CW
GFP
5 µm
0.5h GFP-Bgs3
1.0h GFP-Bgs3
1.5h GFP-Bgs3
2.0h GFP-Bgs3
2.5h GFP-Bgs3

Figure S3

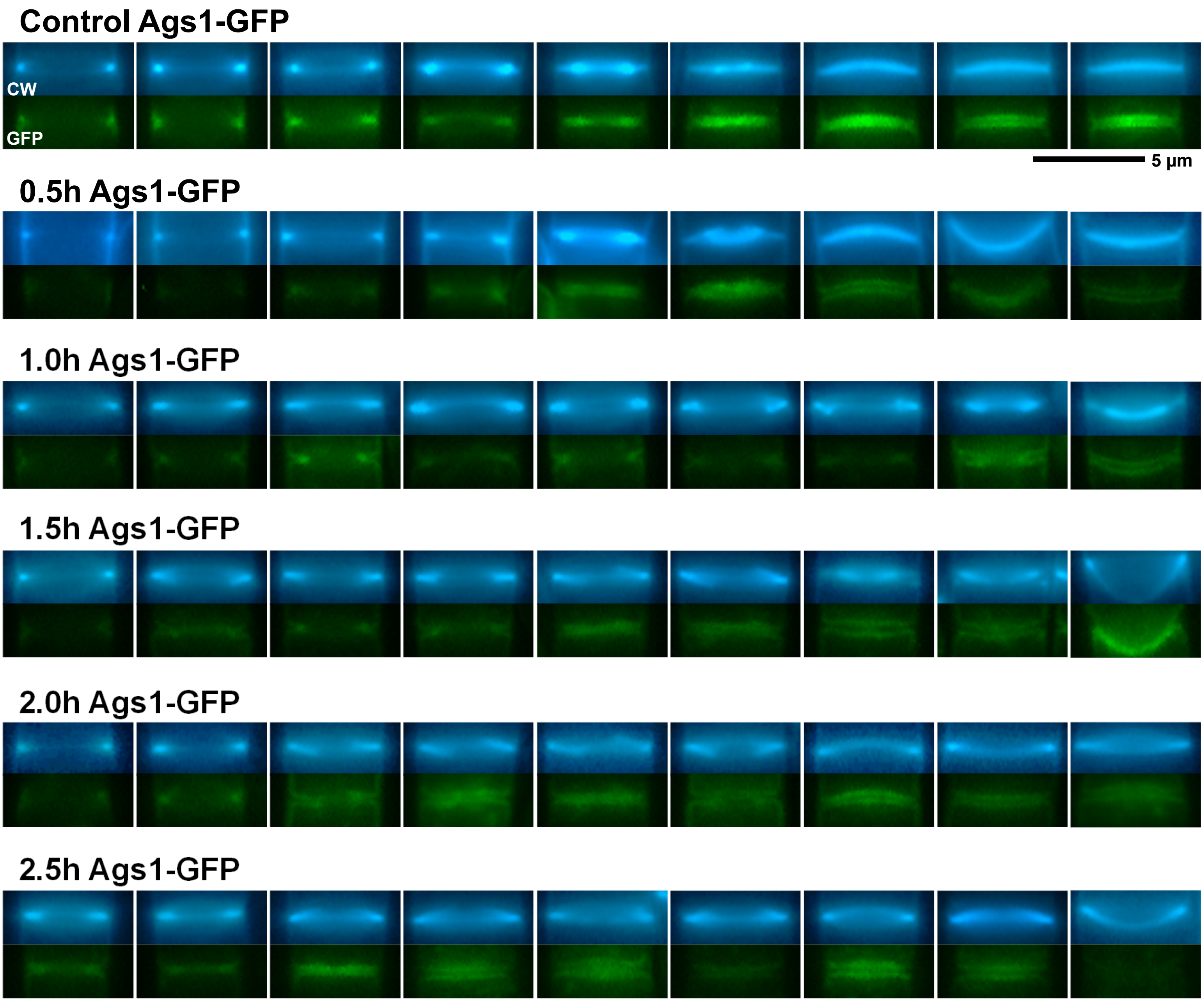
Control Ags1-GFP
CW
GFP
5 µm
0.5h Ags1-GFP
1.0h Ags1-GFP
1.5h Ags1-GFP
2.0h Ags1-GFP
2.5h Ags1-GFP

Figure S4
